# Vorinostat synergizes with ridaforolimus and abrogates the ridaforolimus-induced activation of AKT in synovial sarcoma cells

**DOI:** 10.1186/1756-0500-7-812

**Published:** 2014-11-18

**Authors:** Sherif S Morgan, Lee D Cranmer

**Affiliations:** The University of Arizona Cancer Center, 1515 N. Campbell Avenue, Tucson, AZ 85724-5024 USA

**Keywords:** Synovial sarcoma, Soft-tissue sarcoma, mTOR inhibitor, AKT, Histone deacetylase inhibitor

## Abstract

**Background:**

Curative treatments for patients with metastatic synovial sarcoma (SS) do not exist, and such patients have a poor prognosis. We explored combinations of molecularly-targeted and cytotoxic agents to identify synergistic treatment combinations in SS cells.

**Methods:**

Two SS cell lines (HS-SY-II and SYO-I) were treated with single agents or combinations of molecularly targeted therapies (HDAC inhibitor, vorinostat; mTOR inhibitor, ridaforolimus) and cytotoxic agents. After 72 hours, cell viability was measured using the MTS cell proliferation assay. Combination Indices (CI) were calculated to determine whether each combination was synergistic, additive, or antagonistic. Western Blot analysis assessed alterations in total and phospho-AKT protein levels in response to drug treatment.

**Results:**

We determined the single-agent IC_50_ for ridaforolimus, vorinostat, doxorubicin, and melphalan in HS-SY-II and SYO-I. Synergism was apparent in cells co-treated with ridaforolimus and vorinostat: CI was 0.28 and 0.63 in HS-SY-II and SYO-I, respectively. Ridaforolimus/doxorubicin and ridaforolimus/melphalan exhibited synergism in both cell lines. An additive effect was observed with combination of vorinostat/doxorubicin in both cell lines. Vorinostat/melphalan was synergistic in HS-SY-II and additive in SYO-I. Western blot analysis demonstrated that ridaforolimus increased pAKT-ser473 levels; this effect was abrogated by vorinostat co-treatment.

**Conclusions:**

The combination of ridaforolimus and vorinostat demonstrates *in vitro* synergism in SS. Addition of vorinostat abrogated ridaforolimus-induced AKT activation. Since AKT activation is a possible mechanism of resistance to mTOR inhibitors, adding vorinostat (or another HDAC inhibitor) may be a route to circumvent AKT-mediated resistance to mTOR inhibitors.

## Background

Soft-tissue sarcomas (STS) are a relatively rare and heterogeneous group of malignancies. In the United States, an estimated 11,410 new cases and 4,390 deaths were anticipated in 2013 from STS [1]. Based on Surveillance Epidemiology End Results (SEER) data from 2006 to 2010, synovial sarcoma (SS) accounted for 5% of STS cases [2]. Unresectable or metastatic disease occurs in approximately 40-60% of STS patients [3, 4]. Recurrence rates for SS are high. At 5 years, the risk of recurrence is approximately 12% locally and 39% at distant sites [5]. Metastatic SS portends a poor prognosis, with a median survival of 22 months [6].

The majority of SS cases (95%) are characterized by a fusion between the *SS18* (previously known as *SYT*) gene on chromosome 18 and one of several *SSX* genes (*SSX1*, *SSX2*, or *SSX4*) on the X chromosome [7, 8]. The resulting oncoproteins, particularly SS18-SSX2, are sufficient to promote tumorigenesis [9–11]. Although the exact mechanisms of oncogenesis by the fusion protein remain poorly defined, several studies have implicated SS18-SSX in aberrant transcriptional regulation, chromatin remodeling, and epigenetic gene silencing [12–15]. In addition, *SS18-SSX2* induces *Bcl2* transcription, but represses other anti-apoptotic genes (including *Mcl1* and *Bcl2a1*) [16]. Overexpression of BCL2 is characteristic of SS [17–19] and has been previously explored as a potential therapeutic target. In FU-SY-1, an SS cell line, knockdown of BCL2 using an antisense oligonucleotide sensitized cells to the cytotoxic effects of doxorubicin [20]. Nevertheless, our understanding of SS pathogenesis has yet to yield guidance for molecularly targeted therapy.

Treatment of unresectable or metastatic SS relies on a limited number of cytotoxic agents [21]. Responses are not typically durable and most patients will require salvage therapy. Targeted therapies may hold promise in the treatment of SS, but our understanding of the context and the biology of molecular targets remain critical. The mere presence of a molecular target does not indicate *ipso facto* that it is involved in the initiation or progression of a disease. For example, a recent phase 2 study in SS patients failed to demonstrate positive activity of gefitinib even though patients were selected based on their HER-1 expression status [22]. This result highlights the importance of understanding the biology of the disease in application of targeted therapy approaches.

Given the previously reported effects of SS18-SSX on epigenetic gene silencing [12–15] and the significance of the AKT signaling pathway in SS [23], we sought to determine the effects of vorinostat (HDAC inhibitor) and ridaforolimus (mTOR inhibitor) as single agents, in combination with each other, and in combination with cytotoxic chemotherapies commonly used to treat SS.

## Methods

### Cell culture

HS-SY-II and SYO-I were provided by A. Kawai (National Cancer Center Hospital, Tokyo, Japan) and M. Ladanyi (Memorial Sloan Kettering Cancer Center, New York, NY), respectively. Cell lines were authenticated using short tandem repeat (STR) analysis. Cells were maintained in RPMI1640 medium (Mediatech; Herndon, VA) supplemented with 10% fetal bovine serum (Atlas Biologicals, Fort Collins, CO) and cultured at 37°C in a humidified and 5% CO_2_ atmosphere.

### Drugs

Both vorinostat and ridaforolimus were provided by Merck (Whitehouse Station, NJ). Doxorubicin and melphalan were obtained from Sigma-Aldrich (St. Louis, MO). All drugs were stored as 10 mM stock solutions. Vorinostat was dissolved in DMSO, ridaforolimus in ethanol, doxorubicin in sterile water, and melphalan in ethanol containing 0.5% HCl.

### Cell viability assay

Cells were seeded in quadruplicate in 96-well plates at a density of 4.0 × 10^3^ cells per well for 24 hours, followed by incubation with vehicle control or drug(s) for 72 hours. All control and experimental wells received equivalent concentration of vehicle. MTS reagent (CellTiter 96® AQ_ueous_ One Solution Cell Proliferation Assay; Promega) was added to each well directly into the culture medium and incubated at 37°C for 3 hours in a humidified, 5% CO_2_ atmosphere, as described in the kit’s instructions. Following incubation, absorbance was recorded at wavelength of 490 nm.

### Calculation of IC_50_

We determined the IC_50_, defined as the concentration of drug needed to decrease cell viability by 50%, for each agent alone and in combination with other agents. To determine IC_50_, cell viability was measured in response to a series of 6 drug concentrations; starting with the smallest, each subsequent concentration was doubled. The dose–response curve for each agent was plotted (drug concentrations on the x-axis and % of viable cells on the y-axis ranging from 0 to 1). Linear regression was conducted and IC_50_ was estimated using the following equation, derived from the fitted line (Y = aX + b):


### Calculation of combination index (CI) values

To determine whether a combination of drugs is synergistic, additive, or antagonistic, cells were treated with multiples of each drug’s IC_50_. CI was calculated using the median-effect analysis method of Chou and Talalay [24, 25], as described below:


where D_1_ and D_2_ are doses of drugs 1 and 2 that have x effect when used in combination, and (D_x_)_1_ and (D_x_)_2_ are doses of drugs 1 and 2 that have the same x effect when used alone as single agents. In our study, x was defined as the IC_50_. The Chou and Talalay method was developed as a result of more than 40 years of research, resulting in the introduction of “combination index” to quantitatively express effects of drug combinations [26]. When compared to other methods in evaluating drug combination effects, CI results led to the same conclusions as other methods did [27]. Taken together, CI is widely used and accepted as a reliable method to analyze the interactions and effects of drug combinations.

### Western blot analysis

Cells were seeded in 6-well plates at a density of 0.5 × 10^6^ per well for 24 hours, followed by incubation with vehicle control, ridaforolimus (15 nM), vorinostat (500 nM), or their combination for 72 hours. Cells were rinsed with PBS, then scraped in lysis buffer (Cell Signaling Technology; Danvers MA), supplemented with protease and phosphatase inhibitor cocktails (Roche Applied Science; Indianapolis, IN). Lysates were centrifuged for 5 minutes at 13,000 × *g* and quantified using the Protein Assay Reagent from Bio-Rad (Hercules, CA). Equal amounts of proteins (35 μg) were loaded onto 10% NuPage gels (Invitrogen; Carlsbad, CA). Following transfer, blocking, and incubation with primary and secondary antibodies, proteins were visualized by electrochemiluminescence (Perkin-Elmer; Boston, MA) and exposed to HyBlot CL films (Denville Scientific; Metuchen, NJ). Phospho-serine 473 AKT and total AKT antibodies were obtained from Cell Signaling Technology (Danver, MA). β-actin was purchased from Sigma-Aldrich (St. Louis, MO). Secondary antibodies coupled to horseradish peroxidase were obtained from GE HealthCare (Piscataway, NJ). Densitometric semi-quantification of bands was conducted using the ImageJ software (National Institutes of Health). Levels of p-AKT-ser473 bands were normalized to their corresponding total-AKT bands, which were normalized to their corresponding β-actin bands.

### Statistical analysis

All data are presented as means ± standard errors of the means (SE) obtained from at least three independent experiments. To determine statistical significance of differences observed, Student’s *t*-tests were performed with *p* < 0.05 being considered significant.

## Results

### Sensitivity of cells to ridaforolimus, vorinostat, and cytotoxic agent

**Figure 1:**
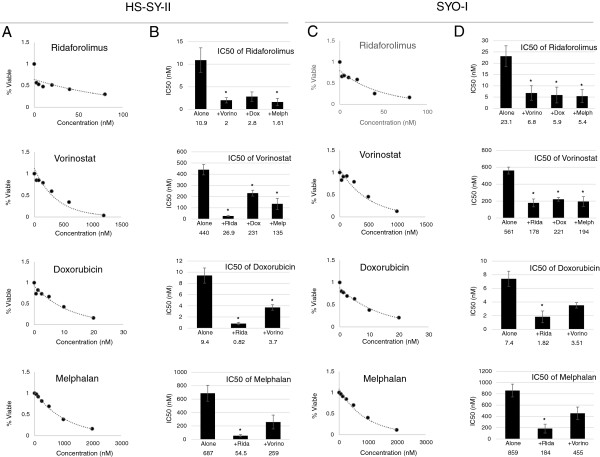
**Cell proliferation assay (MTS) to determine IC**_**50**_ **of single agent and combination treatments in HS-SY-II and SYO-I. A** and **C**, representative dose–response curves of HS-SY-II **(A)** and SYO-I **(C)** treated with ridaforolimus, vorinostat, doxorubicin, and melphalan. **B** and **D**, graphs representing the mean ± SE IC_50_ values for single agents and combination treatments in HS-SY-II **(B)** and SYO-I **(D)** from at least 3 independent experiments. *Indicates statistical significance of *p* < 0.05.

To obtain baseline information regarding the sensitivity of HS-SY-II and SYO-I to the agents of interest, cells were treated with molecularly targeted therapies (HDAC inhibitor, vorinostat; mTOR inhibitor, ridaforolimus) and cytotoxic agents (doxorubicin and melphalan) as single agents. After 72 hours, cell viability was measured as described in the methods section. The mean IC_50_s of each agent in both cell lines are summarized in Table 1. We conducted *t*-tests to determine if the relative sensitivities of the two cell lines for a given drug are statistically significant. HS-SY-II is more sensitive to ridaforolimus than SYO-I in a statistically significant manner (*p* < 0.05). There were no other statistically significant differences in relative sensitivities between the two cell lines. Figure 1 shows sample dose–response curves for each agent in HS-SY-II (Figure 1A) and SYO-I (Figure 1C).

**Table 1:** **IC**_**50**_ **in nM of single agent treatments in HS-SY-II and SYO-I**

Treatments	HS-SY-II	SYO-I
Ridaforolimus	10.9 ± 2.7 nM*	23.1 ± 4.6 nM*
Vorinostat	440 ± 46.7 nM	561 ± 40.4 nM
Doxorubicin	9.4 ± 1.4 nM	7.4 ± 1.1 nM
Melphalan	687 ± 117.6 nM	859 ± 113.7 nM

Data presented in Table 1 represent the mean ± SE from at least 8 independent experiments.

*Indicates statistical significance of *p* < 0.05, when the IC_50_ of a given agent was compared between the two cell lines.

### Combination treatment effects

HS-SY-II and SYO-I were co-treated with ridaforolimus and vorinostat. In addition, cells were treated with each cytotoxic drug (doxorubicin and melphalan) in combination with either ridaforolimus or vorinostat. Cell viability was measured after 72 hours. The IC_50_ values of each agent alone and in combination are represented in Figures 1B (HS-SY-II) and 1D (SYO-I). In HS-SY-II, the IC_50_ of ridaforolimus alone was 10.9 ± 2.7 nM, which was decreased to 2 ± 0.6 nM when combined with vorinostat (*p* < 0.05), 2.8 ± 1.1 nM with doxorubicin, and 1.6 ± 0.8 nM with melphalan (*p* < 0.05). In HS-SY-II, the IC_50_ of vorinostat alone was 440 ± 46.7 nM, which was decreased to 26.9 ± 6.5 nM when combined with ridaforolimus (*p* < 0.05), 231 ± 26.4 nM with doxorubicin (*p* < 0.05), and 135 ± 49.7 nM with melphalan (*p* < 0.05). In HS-SY-II, the IC_50_ of doxorubicin alone was 9.4 ± 1.4 nM, which was decreased to 0.82 ± 0.2 nM when combined with ridaforolimus (*p* < 0.05) and 3.7 ± 0.5 nM with vorinostat (*p* < 0.05). In HS-SY-II, the IC_50_ of melphalan alone was 687 ± 117.6 nM, which was decreased to 54.5 ± 16.9 nM when combined with ridaforolimus (*p* < 0.05) and 259 ± 103.4 nM with vorinostat. In SYO-I, the IC_50_ of ridaforolimus alone was 23.1 ± 4.6 nM, which was decreased to 6.8 ± 3.2 nM when combined with vorinostat (*p* < 0.05), 5.9 ± 3.5 nM with doxorubicin (*p* < 0.05), and 5.4 ± 2.9 nM with melphalan (*p* < 0.05). In SYO-I, the IC_50_ of vorinostat alone was 561 ± 40.4 nM, which was decreased to 178 ± 46.6 nM when combined with ridaforolimus (*p* < 0.05), 221 ± 21.9 nM with doxorubicin (*p* < 0.05), and 194 ± 57.8 nM with melphalan (*p* < 0.05). In SYO-I, the IC_50_ of doxorubicin alone was 7.4 ± 1.1 nM, which was decreased to 1.82 ± 0.86 nM when combined with ridaforolimus (*p* < 0.05) and 3.5 ± 0.4 nM with vorinostat. In SYO-I, the IC_50_ of melphalan alone was 859 ± 113.7 nM, which was decreased to 184 ± 78.3 nM when combined with ridaforolimus (*p* < 0.05) and 455 ± 110.3 nM with vorinostat.

The CI of ridaforolimus/vorinostat was 0.28 ± 0.06 and 0.63 ± 0.14 in HS-SY-II and SYO-I, respectively, indicating synergism between the two agents in both cell lines. The difference in CI of ridaforolimus/vorinostat in the two cell lines was statistically significant (*p* < 0.05). The combinations of ridaforolimus/doxorubicin and ridaforolimus/melphalan also exhibited synergism in both cell lines (CI ranged from 0.50 to 0.59). Additive effects were observed when vorinostat was combined with doxorubicin in both cell lines (CIs were 1.1 ± 0.06 in HS-SY-II and 0.98 ± 0.11 in SYO-I). Vorinostat/melphalan was synergistic in HS-SY-II (CI 0.81 ± 0.08), but additive in SYO-I (0.90 ± 0.02). However, the difference in CI values of vorinostat/melphalan between the two cell lines was not statistically significant. The CIs for all drug combination are summarized in Table 2.

**Table 2:** **Combination Indices (CI) for combination treatments in HS-SY-II and SYO-I**

HS-SY-II			SYO-I		
	Ridaforolimus	Vorinostat		Ridaforolimus	Vorinostat
Vorinostat	0.28 ± 0.06*	n/a	Vorinostat	0.63 ± 0.14*	n/a
Doxorubicin	0.56 ± 0.13	1.1 ± 0.06	Doxorubicin	0.50 ± 0.10	0.98 ± 0.11
Melphalan	0.51 ± 0.06	0.81 ± 0.08	Melphalan	0.59 ± 0.17	0.90 ± 0.02

Data presented in Table 2 represent the mean ± SE from at least 3 independent experiments.

*Indicates statistical significance of *p* < 0.05, when the CI for a given combination was compared between the two cell lines.

Because of the synergistic activity of the ridaforolimus/vorinostat combination, other cell lines representing various tumor types were assessed, including osteosarcoma (U2OS), metastatic melanoma (Stew1 and Stew2), pancreatic cancer (Panc1 and BxPC3), and lung cancer (A549). The combination was synergistic in all cell lines tested (CI ranged from 0.37 to 0.77), except in Panc1, where it was additive (CI was 0.92) (data not shown).

### Effect of vorinostat on ridaforolimus-induced phosphorylation of AKT

**Figure 2:**
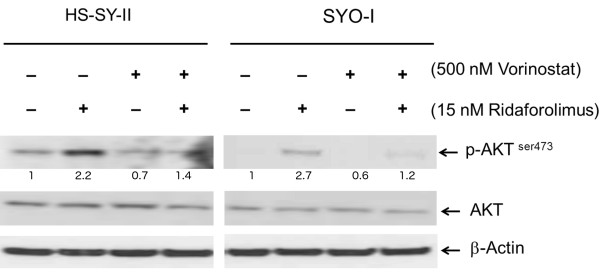
**Effects of ridaforolimus and vorinostat on AKT in HS-SY-II and SYO-I.** Representative Western blot analysis of cell lysates of HS-SY-II and SYO-I treated with ridaforolimus (15 nM), vorinostat (500 nM), or their combination for 72 hours. Cell lysates were subjected to immunoblot analysis with phospho-AKT-ser473, total AKT, and β-actin antibodies. This experiment was undertaken twice. Semi-quantification of p-AKT-ser473 is depicted under each band. Levels of p-AKT-ser473 bands were normalized to their corresponding total-AKT bands, which were normalized to their corresponding β-actin bands.

We sought to determine if ridaforolimus would increase the levels of phosphorylated AKT. HS-SY-II and SYO-I were treated with vorinostat (500 nM), ridaforolimus (15 nM), or their combination for 72 hours. These concentrations were approximately those that achieved the IC_50_ in the *in vitro* viability assay (refer to Table 1). Cells were harvested and subjected to immunoblot analysis for phospho-AKT-serine473 (p-AKT-ser473). As expected, ridaforolimus treatment increased levels of p-AKT-ser473 in both HS-SY-II and SYO-I, while vorinostat alone exerted no significant effect on p-AKT-ser473 levels as compared to vehicle-treated cells (Figure 2). In both cell lines, co-treatment with ridaforolimus and vorinostat suppressed p-AKT-ser473 levels compared to the elevated levels seen when cells were treated with ridaforolimus alone. Semi-quantification of p-AKT-ser473 is depicted in Figure 2.

## Discussion

We evaluated the effects of a number of cytotoxic and molecularly-targeted agents on the viability of two SS cell lines, HS-SY-II and SYO-I. Using an *in vitro* cell viability assay, we observed a synergism in both HS-SY-II and SYO-I when cells were co-treated with ridaforolimus and vorinostat. Synergism was also observed in HS-SY-II and SYO-I when ridaforolimus was combined with doxorubicin or melphalan. Combination of vorinostat with doxorubicin yielded additive effects in both cell lines, while combination of vorinostat with melphalan was synergistic in HS-SY-II and additive in SYO-I.

Our *in vitro* data demonstrating synergistic and additive effects with combination of either ridaforolimus or vorinostat with cytotoxic chemotherapies (*e.g.*, doxorubicin) may have clinical relevance. Ridaforolimus or vorinostat may serve as chemotherapy-sparing agents by reducing the dose of the cytotoxic therapies needed to achieve similar or better tumor control. Doing so would potentially delay or prevent dose-limiting toxicities. This may be especially relevant in the case of doxorubicin, a backbone therapy for STS, but one that is hampered by dose-limiting cardiotoxicity.

Previously, others have shown that several mTOR inhibitors induced activation of AKT through a negative feedback, which may be partially responsible for developing mTOR inhibitor resistance [28, 29]. In our studies, ridaforolimus induced the activation of AKT in both SS cell lines. The addition of vorinostat abrogated the ridaforolimus-induced activation of AKT in both SS cell lines. A recent study demonstrated that HDAC 3, which belongs to the HDAC class I protein family, is necessary for activation of the AKT/mTOR pathway [30]. Since vorinostat inhibits HDAC class I and II enzymes [31, 32], it is possible that vorinostat attenuates ridaforolimus-induced activation of AKT through its inhibition of HDAC 3. Other studies, however, are needed to explain how vorinostat attenuates the ridaforolimus-mediated activation of AKT in HS-SY-II and SYO-I.

HDACs are involved in modulating several signaling pathways and cellular processes [33]. Inhibition of HDAC by vorinostat has been shown to induce cell cycle arrest, autophagy, and apoptosis [34–39]. These effects are mediated, at least partially, through vorinostat’s effects on several signaling pathways, including AKT, mTOR, MAPK, JAK-STAT, NF-κB, and others [33, 40–45]. The vorinostat-mediated effects on these signaling pathways likely contribute to the synergism observed between ridaforolimus and vorinostat. Of interest, in renal cell cancer, vorinostat enhanced the activity of temsirolimus by suppressing levels of *survivin*, a member of the inhibitor of apoptosis (IAP) gene family [46]. Transcriptional regulation of *survivin* is complex, mediated by cell cycle dependent mechanisms as well as stimulation by growth factor and cytokines [47]. Levels of *survivin* mRNA is stabilized via the mTOR pathway upon stimulation with insulin-growth factor [48, 49], which may explain how mTOR inhibition sensitizes cells to apoptosis. Future investigations will focus on understanding the underlying mechanisms responsible for the synergy between ridaforolimus and vorinostat.

## Conclusions

Our preliminary findings demonstrate an *in vitro* synergy between ridaforolimus and vorinostat in SS cells. Although further investigations are necessary, this combination may have a broad anti-neoplastic activity in a variety of tumor types. We also found that addition of vorinostat abrogates the ridaforolimus-induced AKT activation. Since AKT activation is a possible mechanism of resistance to mTOR inhibitors, adding vorinostat (or another HDAC inhibitor) may be a route to circumvent AKT-mediated resistance to mTOR inhibitors.

## Abbreviations

CI: Combination index
HDAC: Histone deacetylase
IAP: Inhibitor of apoptosis
mTOR: Mammalian target of rapamycin
SE: Standard errors of the means
SEER: Surveillance Epidemiology End Results
SS: Synovial sarcoma
STR: Short tandem repeats
STS: Soft-tissue sarcoma.
